# A novel cardiovascular magnetic resonance risk score for predicting mortality following surgical aortic valve replacement

**DOI:** 10.1038/s41598-021-99788-7

**Published:** 2021-10-12

**Authors:** Vassilios S. Vassiliou, Menelaos Pavlou, Tamir Malley, Brian P. Halliday, Vasiliki Tsampasian, Claire E. Raphael, Gary Tse, Miguel Silva Vieira, Dominique Auger, Russell Everett, Calvin Chin, Francisco Alpendurada, John Pepper, Dudley J. Pennell, David E. Newby, Andrew Jabbour, Marc R. Dweck, Sanjay K. Prasad

**Affiliations:** 1 CMR Unit, Department of CMR, Royal Brompton Hospital and National Heart and Lung Institute, Imperial College, Sydney Street, London, SW3 6NP UK; 2 Department of Cardiology, Norwich Medical School, University of East Anglia, Norfolk and Norwich University Hospital, Floor 2, Bob Champion Building, James Watson Road, Norwich, NR4 7UQ UK; 3 Department of Statistical Science, University College London, London, UK; 4 Kent and Medway Medical School, Canterbury, UK; 5 Second Hospital of Tianjin, Medical University, Kent, China; 6 Centre for Cardiovascular Sciences, University of Edinburgh, Edinburgh, USA; 7 Department of Cardiology, National Heart Centre, Singapore, Singapore; 8 Department of Cardiology, St. Vincent’s University, Sydney, NSW Australia

**Keywords:** Cardiology, Interventional cardiology, Risk factors

## Abstract

The increasing prevalence of patients with aortic stenosis worldwide highlights a clinical need for improved and accurate prediction of clinical outcomes following surgery. We investigated patient demographic and cardiovascular magnetic resonance (CMR) characteristics to formulate a dedicated risk score estimating long-term survival following surgery. We recruited consecutive patients undergoing CMR with gadolinium administration prior to surgical aortic valve replacement from 2003 to 2016 in two UK centres. The outcome was overall mortality. A total of 250 patients were included (68 ± 12 years, male 185 (60%), with pre-operative mean aortic valve area 0.93 ± 0.32cm^2^, LVEF 62 ± 17%) and followed for 6.0 ± 3.3 years. Sixty-one deaths occurred, with 10-year mortality of 23.6%. Multivariable analysis showed that increasing age (HR 1.04, *P* = 0.005), use of antiplatelet therapy (HR 0.54, *P* = 0.027), presence of infarction or midwall late gadolinium enhancement (HR 1.52 and HR 2.14 respectively, combined *P* = 0.12), higher indexed left ventricular stroke volume (HR 0.98, *P* = 0.043) and higher left atrial ejection fraction (HR 0.98, *P* = 0.083) associated with mortality and developed a risk score with good discrimination. This is the first dedicated risk prediction score for patients with aortic stenosis undergoing surgical aortic valve replacement providing an individualised estimate for overall mortality. This model can help clinicians individualising medical and surgical care.

*Trial Registration* ClinicalTrials.gov Identifier: NCT00930735 and ClinicalTrials.gov Identifier: NCT01755936.

## Introduction

Aortic valve stenosis (AS) is the most common valvular heart disease in the Western world, characterised by progressive narrowing of the valve^1,2^ and a prevalence expected to double over the next 20 years as a result of an aging population^3^. Currently, AS is the most common condition necessitating valve replacement surgery worldwide, representing a major source of global morbidity and mortality and poses a substantial burden on healthcare resources^4^. Despite this, there is currently no dedicated risk score derived specifically for patients undergoing surgical aortic valve replacement (SAVR) for estimating long-term mortality to allow clinicians to facilitate precision medicine.

At present, both North American and European guidelines^5,6^ recommend surgery in symptomatic patients with severe AS and sometimes even in asymptomatic patients with evidence of LV decompensation defined usually as a reduction in LV ejection fraction (LVEF < 50% attributed to AS). However, several studies have shown that other more objective parameters of LV decompensation are independently associated with worse short-to-medium term outcomes, including identification of myocardial fibrosis on histology^7,8^ and fibrosis identified via late-gadolinium enhancement cardiovascular magnetic resonance (LGE-CMR). Indeed the presence of midwall LGE in AS is associated with an adverse prognosis in single centre^9–11^, and one multi-centre study^12^. However, even after successful AVR, mortality remains high and Euroscore II and STS models whilst predicting in-hospital and 30-day mortality, they lack the inclusion of myocardial fibrosis in their models which is a known risk factor. Furthermore, although individual predictors have already been investigated including LVEF and midwall fibrosis, no score is currently available to bring all those parameters together in predicting risk.

In this study we investigated the additional role of LGE-CMR in developing a mortality risk score for patients undergoing SAVR in two institutions, and identified predictors of survival following aortic valve replacement.

## Methods

### Patient population

Consecutive patients with aortic stenosis undergoing LGE-CMR and subsequent SAVR were recruited from two large prospective observational registries: the Royal Brompton Hospital of Imperial College, London, UK including patients from 2003–2016 (ClinicalTrials.gov Identifier: NCT00930735, June 30th 2009) and the Edinburgh Heart Centre, Royal Infirmary of University of Edinburgh, Edinburgh, UK including patients from 2013 to 2016 (ClinicalTrials.gov Identifier: NCT01755936, December 24th 2012). The present study is not associated with the objectives of these trials and does not report results associated with or generated from these trials. It uses clinical data generated from these trials to investigate the specific objective mentioned above. The study was conducted in accordance with the Declaration of Helsinki after local research ethics approval and written patient consent.

### Data collection

Medical history and demographic characteristics were collected following patient interviews and review of the hospital and community records. Coronary artery disease was defined as prior coronary revascularization or the presence of significant coronary artery stenosis as assessed by invasive or computed tomography coronary angiography by > 50% lumen diameter narrowing.

### Cardiovascular magnetic resonance

At the Royal Brompton Hospital, LGE-CMR was undertaken at 1.5 T (Magnetom Sonata or Avanto, Siemens AG, Erlangen, Germany) while at the Edinburgh Heart Centre a 3 T Magnetom Verio (Siemens AG, Erlangen, Germany) was used. A standardised protocol was undertaken in each centre as described previously^13,14^. In brief, after localisers, steady-state free precession sequences were used for aortic valve planimetry (two orthogonal coronal views were taken, and then sagittal “valve stack” imaging starting at ~ 10 mm below the level valve and extending to ~ 10 mm above the level of the valve), and assessment of biventricular volumes and LV mass. Ten to fifteen minutes after injection of 0.1 mmol/kg of gadolinium contrast agent (Gadovist, Schering AG, Berlin, Germany) inversion recovery–prepared spoiled gradient echo images were acquired in standard long- and short-axis views to detect areas of LGE.

### CMR image analysis

Anonymised images were analysed at the Royal Brompton Core laboratory with CMR Tools (Cardiovascular solutions, London, UK)^9,14^. The severity of aortic stenosis was assessed using validated CMR-derived planimetry of the aortic valve area (AVA)^15^, and graded as follows: mild, > 1.5 to 2.5 cm^2^; moderate, 1.5 to 1.0 cm^2^; and severe, < 1.0 cm^2^ in accordance with the American College of Cardiology/American Heart Association guidelines^16^. Left atrial volumes in systole and diastole were obtained by the biplane area-length described previously^17^ and were used to calculate the EF as follows:

$${\text{LA}}\;{\text{ejection}}\;{\text{fraction}} = 100 \times {{\left( {{\text{LA}}\;{\text{diastolic}}\;{\text{volume}} - {\text{LA}}\;{\text{systolic}}\;{\text{volume}}} \right)} \mathord{\left/ {\vphantom {{\left( {{\text{LA}}\;{\text{diastolic}}\;{\text{volume}} - {\text{LA}}\;{\text{systolic}}\;{\text{volume}}} \right)} {{\text{LA}}\;{\text{diastolic}}\;{\text{volume}}}}} \right. \kern-\nulldelimiterspace} {{\text{LA}}\;{\text{diastolic}}\;{\text{volume}}}}$$

### Clinical endpoint

The endpoint was all-cause mortality. This was confirmed from hospital notes, communication with primary care and through the Office of National Statistics, where there is compulsory registration of all deaths.

### Statistical analysis

All statistical analyses were carried out using STATA (14, StataCorp. College Station, TX, USA) and R version 3.2. Variables are expressed as mean ± standard deviation (SD), median and interquartile range (IQR) or counts and percentages as appropriate. The follow-up time for each patient was calculated from the day of CMR to the date of death or their most recent evaluation. The annual event rate was calculated by dividing the number of patients reaching the endpoint by the total follow-up period for that endpoint. The cumulative probability for the occurrence of an outcome was estimated using the Kaplan–Meier method.

### Missing data

No variable had more than 10% of data missing. Nonetheless, multiple imputation was undertaken in variables with any missing data as described in Supplementary Material Methods.

### Model development

Cox regression was used to model the relationship between the outcome and the significant univariate predictors as from previous studies^18–21^. The follow-up time for each patient was time from the date of CMR to death, the end of study period or last follow-up date. Patients alive at the end of study period or who were lost to follow-up were censored. Univariable Cox regression models were fitted for all potential predictors, and those significant at the 10% level were considered in multivariable analysis. A higher threshold than the conventional 5% was chosen to minimise the possibility of excluding variables that are only significant in the presence of others, in line with similar studies^22,23^. These predictors were then fitted in a multivariable model and the final model was derived using forward selection at the 15% significance level, so that the selection process did not start with an overly complicated model, given the relatively small number of events. Backwards elimination was also used as a sensitivity analysis. The degree of model overfitting was assessed and adjusted for using internal validation techniques^24^. The proportional hazards assumption required by the Cox model was investigated using Schoenfeld residuals^23^.

### Model validation

The risk model was validated internally using bootstrap validation (200 bootstrap samples) and measures of predictive performance assessing calibration (calibration slope) and discrimination (Uno’s C-index) were calculated^25^. The calibration slope was used to assess the degree of agreement between the observed and predicted risk of mortality and to adjust for potential model overfitting. Specifically, the estimated regression coefficients were shrunk by a factor equal to the calibration slope estimated from bootstrapping (linear shrinkage factor)^26^ Calibration was also examined using a calibration plot, by comparing the observed and predicted risk of mortality at 10 years in clinically meaningful risk groups (group cut-offs: 0–25, 25–45, 45–60, and > 60% 10-year risk of mortality). The C-index was used to measure how well the model discriminated between patients with high and low risk of death^26^.

### Ethics approval and consent to participate

UK National Ethics approval from London and Lothian were obtained. Institutional Board approval from Edinburgh Royal Infrimary and Royal Brompton Hospital and written informed patient consent were obtained.

### Consent for publication

No individual patient data shown.

## Results

A total of 250 patients (London 211, Edinburgh 39) were included in this study: age 68 ± 12 years, 185 (74%) male, aortic valve area = 0.93 ± 0.32 cm^2^. There were 161 patients with isolated SAVR, while 89 had SAVR and CABG. A total of 168 (67%) patients had severe aortic stenosis while 82 (33%) had moderate aortic stenosis. All the patients with moderate aortic stenosis had a concomitant CABG.

Coronary artery disease was present in 114 (46%) and 37 (15%) patients had low flow (defined by LV stroke volume < 35mls/m^2^) (Table 1). CMR was performed at a median of 56 days before the operation (range 14–184).

**Table 1:** Baseline characteristics of patients according to presence or absence of late gadolinium enhancement on CMR.

	No GAD (n = 113)	Midwall GAD (n = 89)	Infarction GAD (n = 48)	*P*-value	Overall
	Mean or %	Mean or %	Mean or %		Mean or %
Age	66 ± 13	70 ± 12	69 ± 11	0.136	68 ± 12
Sex/ male	66.4	79.8	81.3	0.043	74
BSA	1.87 ± 0.23	2.02 ± 0.21	1.96 ± 0.21	0.000	1.94 ± 0.23
Weight	77 ± 17	84 ± 14	80 ± 18	0.007	80 ± 16
BMI	26 ± 5	29 ± 5	32 ± 5	0.008	28 ± 14
Known history of CAD	33.6	40.4	83.3	0.000	45.6
DM	20.4	22.5	29.2	0.473	22.8
CABG	7.1	16.9	25	0.007	14
PCI	8	11.2	29.2	0.001	13.2
Hypercholesterolaemia	30.1	31.5	54.2	0.009	35.2
CVA	4.4	5.6	14.6	0.055	6.8
CKD	3.5	5.6	6.3	0.689	4.8
AF	19.5	22.5	18.8	0.828	20.4
Aspirin/ clopidogrel	53.1	60.7	68.8	0.164	58.8
Statin	48.7	47.2	68.8	0.034	52
ACEI/ ARB	39.8	47.2	56.3	0.149	45.6
Aldo antagonist	18.6	15.7	12.5	0.620	16.4
Beta blocker	35.4	29.2	43.8	0.230	34.8
Ca channel blocker	15.9	20.2	18.8	0.724	18
Diuretic	23.9	32.6	31.3	0.352	28.4
Digoxin	15.9	5.6	14.6	0.067	12
Warfarin	16.8	14.6	4.2	0.095	13.6
Amiodarone	5.3	6.7	6.3	0.910	6
Creatinine	85 ± 24	92 ± 22	89 ± 23	0.149	88 ± 23
NYHA > 2	8	20.2	19.1	0.028	14.5
Euroscore II	1.8 ± 1.6	2.8 + 2.2	2.6 + 1.7	0.003	2.3 ± 1.9
LVEF	67 ± 14	59 ± 18	53 ± 17	0.000	62 ± 17
RVEF	70 ± 12	59 ± 12	59 ± 10	0.206	60 ± 12
LVEDV index	85 ± 34	87 ± 32	94 ± 31	0.243	88 ± 32
LVESV index	31 ± 28	40 ± 29	47 ± 27	0.001	37 ± 27
LVSV index	55 ± 17	48 ± 16	47 ± 14	0.007	51 ± 16
LV mass index	92 ± 30	103 ± 30	103 ± 27	0.014	98 ± 30
LV Hypertrophy	69.9	87.6	66.7	0.004	75.6
LA volume index	56 ± 32	55 ± 27	55 ± 22	0.967	56 ± 28
LAEF	40 ± 18	35 ± 17	31 ± 15	0.014	37 ± 17
Low flow state	8	23	16.7	0.011	14.9
AVA	0.94 ± 0.35	0.918 ± 0.25	0.921 ± 0.35	0.884	0.93 ± 0.315
LBBB	8.8	12.4	8.3	0.648	10
For redo surgical AVR	14.3	7.9	8.3	0.281	10.8

The patients were followed for a mean 6.0 ± 3.3 years. During this time 61 (24.4%) died, and 4 were lost to follow-up, having moved abroad and censored at the last time known to be alive. The mortality observed was 4.8% at year 1, 9.6% at year 3, 12.5% at year 5 and 23.6% at year 10.

Variables with a *p*-value lower than the 10% significance level were considered in the multivariable analysis, in line with the usual established approach for predictive models^23^.

These factors (Table 2) included patient demographics (including age, sex), symptomatic status (including NYHA classification), prior medical history (including PCI or renal disease), CMR parameters (including LVEF, LV End Systolic Volume indexed (LVSVindex), Right Ventricular Ejection Fraction (RVEF), Left Atrial Ejection Fraction (LAEF), presence of midwall or infarction gadolinium or pharmacotherapy taken (including Angiotensin Converting Enzyme (ACE) inhibitor or Angiotensin Receptor Blockers (ARB); aspirin or clopidogrel). Other variables possibly associated with mortality, including sex (Supplementary Material Fig. 1), BSA (body surface area), prior CABG, DM (diabetes mellitus) or prior SAVR, were not significant.

**Table 2:** Cox proportional hazard model univariable analysis of potential predictors of mortality following SAVR.

Risk factor	HR	*P* value	95% CI
Age	1.060	0.00	1.03–1.08
Euroscore II	1.160	0.00	1.05–1.28
LVSV index	0.960	0.00	0.94–0.98
LVEF	0.980	0.00	0.97–0.99
LAEF	0.970	0.00	0.95–0.98
Low flow state (< 35 ml/m^2^)	2.750	0.00	1.56–4.84
LA systolic volume^a^	1.046	0.01	1.01–1.08
No LGE	1.00	0.012	–
Midwall enhancement	2.11		1.14–3.92
Infarction enhancement	2.53		1.26–5.06
Antiplatelet use	0.550	0.02	0.33–0.92
Creatinine	1.010	0.06	1.00–1.02
RVEF	0.980	0.09	0.96–1.00
LA volume indexed	1.060	0.10	0.9–1.13
PCI	1.710	0.10	0.91–3.23
NYHA > 2	1.640	0.12	0.88–3.04
ACE I/ ARB II blocker	0.670	0.13	0.40–1.13
LA diastolic volume^a^	1.030	0.14	0.99–1.07
LVESV index	1.010	0.15	1.00–1.01
Weight	0.990	0.18	0.97–1.00
BMI	0.970	0.18	0.92–1.02
BSA	0.520	0.22	0.18–1.50
DM	1.410	0.23	0.80–2.47
LV mass index	0.990	0.25	0.99–1.00
Known CAD	1.350	0.25	0.81–2.25
Redo AVR	0.620	0.26	0.26–1.44
Hypertrophy	0.750	0.30	0.43–1.30
AF	1.310	0.35	0.74–2.34
Hypercholesterolaemia	0.780	0.37	0.44–1.35
Beta blocker	1.260	0.38	0.75–2.12
Digoxin	1.380	0.38	0.68–2.80
Known CRF	1.500	0.39	0.60–3.76
LBBB	1.340	0.44	0.63–2.83
Past MI	1.370	0.47	0.59–3.19
Past CABG	1.260	0.49	0.65–2.42
Warfarin	1.230	0.53	0.65–2.32
Aldosterone antagonist	0.820	0.59	0.40–1.67
Valve ring size	0.970	0.63	0.86–1.09
Ca^2+^ channel blocker	1.180	0.63	0.61–2.28
LVEDV index	1.000	0.69	0.99–1.01
AVA	0.900	0.82	0.38–2.14
Statin	1.040	0.87	0.62–1.75
Amiodarone	0.960	0.94	0.35–2.66
CVA	1.020	0.96	0.41–2.56
Male	1.000	0.99	0.56–1.77
Diuretic	1.000	0.99	0.55–1.79

A stepwise forward selection was used, allowing us to investigate multiple variables. From these we identified the six variables showing the strongest prediction which we included in the final model. This model showed only mild overfitting which was nonetheless adjusted for.

^a^Per 10-unit increase.

**Figure 1:**
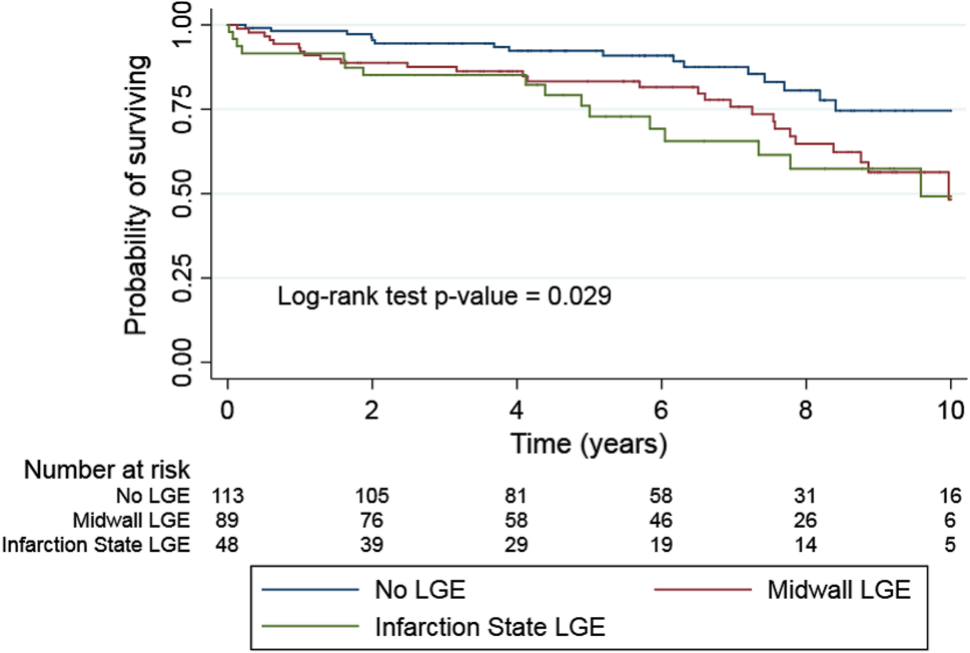
Kaplan–Meier estimator plot of survival in patients with no gadolinium enhancement, midwall enhancement and infarction pattern enhancement. This plot indicates significantly worse prognosis in the patients with either form of enhancement (midwall or infarction) out to 10 years (log rank *P* = 0.029). Patients with a mixed pattern of LGE were categorized according to the predominant pattern of fibrosis.

The presence of either midwall fibrosis or infarction pattern fibrosis was associated with worse outcome when compared to absence of fibrosis (Fig. 1).

**Figure 2:**
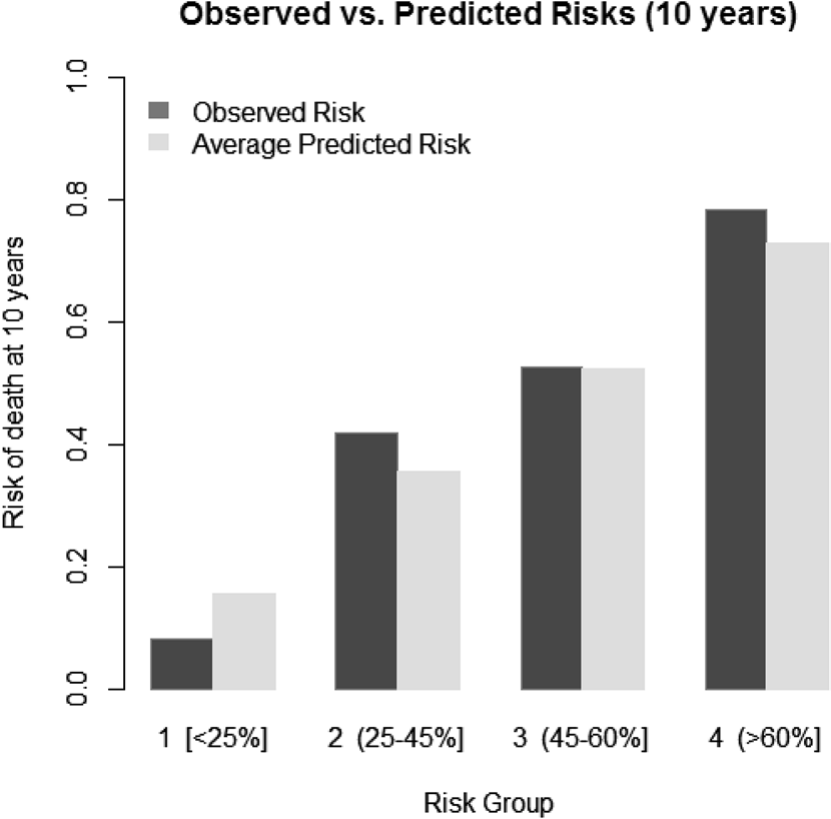
Observed vs predicted risk of mortality for patients following SAVR. The observed (black) vs predicted (grey) risk of mortality for patients following SAVR out to 10 years in clinically relevant risk groups is shown, indicating good prediction for the model. Number of patients per risk group: 73, 94, 42 and 41 for risk groups 1, 2, 3 and 4, respectively.

The variable selection procedure resulted in a final model with five predictors (Table 3): LVSV index (higher LVSV better survival), age (higher age worse survival), use of antiplatelet therapy (aspirin or clopidogrel) prior to SAVR (use associated with better survival), LAEF (higher LAEF associated with better survival) and the presence of myocardial scar (midwall or infarction fibrosis, presence of either associated with worse survival). In internal validation, the model demonstrated satisfactory predictive performance indicating that all five predictors were important. The calibration slope was 0.86 (95% CI = 0.57–1.14), indicating only mild model overfitting, which was nonetheless adjusted for in the prediction equation by multiplying the estimated coefficients (which correspond to the HRs in Table 3) by the shrinkage factor of 0.86. The calibration plot (Fig. 2) shows a good agreement between observed and predicted risks of death at 10 years. The model demonstrated good discrimination with a C-index of 0.72 (95% CI = 0.66, 0.79) compared to the C-index obtained using Euroscore II (C-index 0.66; 95% CI = 0.59, 0.73).

**Table 3:** Multivariable analysis of the strongest predictors.

Multivariable analysis				
Risk factor	HR	*P*-value	95% CI	
LVSV index	0.979	0.043	0.959	0.999
Age	1.043	0.005	1.013	1.073
Antiplatelet use	0.536	0.027	0.309	0.932
No LGE	1.000	0.120	–	–
Midwall LGE	1.520		0.789	2.928
Infarction LGE	2.147		1.035	4.455
LAEF	0.985	0.083	0.969	1.002

Multivariable analysis indicating prognostic variables.

### Prediction equation

The predictions for the risk of death at 10 years can be obtained by the following equation:

$${\text{P}}\left( {{\text{death}}\;{\text{at}}\;{10}\;{\text{yrs}}} \right) = 1 - \left( {0.8761282} \right)^{{\exp \left( {{\text{risk}}\;{\text{score}}} \right)}}$$

where

$$\begin{aligned} {\text{risk}}\;{\text{score}} = & {\text{age}}\;{\text{in}}\;{\text{years}} \times 0.03582 + {\text{aspirin}}/{\text{clopidogrel}} \times \left( { - 0.53552} \right) + {\text{midwall}}\;{\text{LGE}} \times \left( {0.36025} \right) \\ & \quad + {\text{Infarction}}\;{\text{LGE}} { \times } \left( {0.65716} \right) + {\text{LVSV}}\;{\text{index}}\left[ {{\text{mls}}/{\text{m}}^{2} } \right] \times \left( { - 0.01842} \right) + {\text{LAEF}}\left[ \% \right]*\left( { - 0.01278} \right) \\ \end{aligned}$$

and aspirin/clopidogrel, midwall LGE, and infarction LGE are assigned the value 1 if present or zero if absent.

## Discussion

The number of patients with aortic stenosis undergoing SAVR is increasing world-wide, despite of use of TAVR, yet there is no dedicated risk stratification tool to enable precision medicine for those patients. This is the first dedicated multicentre registry determining a risk score utilising myocardial tissue characterisation by CMR and LA function. We confirm the known association of midwall fibrosis with adverse prognosis^9,12^ and further propose that even after successful SAVR overall mortality is high, emphasising the importance of regular review and medical optimisation following surgery.

It is noticeable that our study demonstrated SV, rather than LVEF, to be one of the strong predictors of outcome in this cohort of patients with moderate and severe aortic stenosis. This finding is in agreement with other studies focusing on this specific population cohort^27,28^. Growing literature evidence suggest that in patients with aortic stenosis, it is the longitudinal function of the left ventricular myocardium that becomes significantly impaired. This pathophysiological process may progress with an apparently preserved LVEF, hence other parameters have been used to identify subtle myocardial impairment, including mitral annular plane systolic excursion (MAPSE) and global longitudinal strain (GLS)^29–31^.

Although generic models for predicting survival after open heart surgery exist and are used in clinical practice to identify high risk patients, these are not designed specifically for SAVR and importantly are not specific for estimating long-term survival. A tool for estimating long-term survival following SAVR is therefore needed to enable individualised decisions for patients. We have developed and internally validated a risk score using the most significant variables that can be used to identify patients at risk of overall mortality after SAVR. We have used a pragmatic cohort of patients undergoing routine guideline-based surgery to ensure our findings are clinically relevant reflecting the routine patient demographics. The aim of this work was not to identify the correct surgical “window” for patients with AS, but to identify pre-operative predictors of survival. As such, a model looking at overall mortality in patients who have undergone surgical SAVR based on the existing guidelines is able to identify patients with a high risk of mortality. High-risk patients may benefit from more frequent medical care by physicians and cardiologists. This model will also allow clinicians to consider longer-term outcomes in patients, as currently the use of Euroscore II and STS only allows short-term outcome prediction.

One important novel finding is the prognostically beneficial use of antiplatelet therapy. In the UK if patients take antiplatelet therapy before surgery this is continued long-term unless anticoagulation is needed. Multivariable analysis showed use of antiplatelets was associated with an almost 50% reduction in overall mortality, independently of presence or absence of coronary disease. This suggests that patients with AS undergoing SAVR may represent a cohort of patients at high vascular risk who might benefit from antiplatelet therapy in the long term. As our cohort of patients was elderly and the vast majority received a tissue bioprosthesis (> 90%) we estimate that the continuation of aspirin or clopidogrel could also have had an impact in reducing tissue thrombosis and hence improve survival.

### Clinical implications

We provide a validated score with predictive variables for calculating mortality risk out to 10 years. This score can be used, to identify patients at higher risk following the SAVR that could benefit from being followed up in the hospital cardiology outpatients or the community more closely. More specifically, this score is applicable not only to the patients with severe AS undergoing SAVR but also to the patients with moderate AS and co-existent CAD undergoing SAVR and CABG. Importantly, as our model is derived from prognostically important risk factors, it subsequently enables early identification of the patients that carry high risk of mortality post intervention. We did not compare AVR vs. medical management. Therefore, even if the risk might be high, the individual patient might still fare much better with surgery than medical management. Therefore, the aim of the score is to facilitate more tailored post-operative management, than act as prohibitive to surgery, or indeed TAVR, as this falls out of the scope and purpose of the model. Furthermore, following appropriate validation, this score could be routinely utilised for selecting patients for transcatheter aortic valve replacement in preference to the non-specific Euroscore II and STS, although evidence from randomised controlled trials would be invaluable in validating this. Finally, our results indicate that use of an antiplatelet at the time of CMR is associated with significantly improved mortality. This is a novel finding suggesting SAVR patients represent a cohort with high vascular risk, including valve thrombosis, that might benefit from antiplatelet therapy independently from other comorbidities. This work lends support to this hypothesis and further studies will be needed, however, to determine antiplatelet duration.

### Study limitations

Even with two centres, referral bias is possible. However, all our patients fulfilled a clinical indication for SAVR and our catchment areas for referrals is large, spanning across the UK. Additionally, the period of patient enrolment was different between the two centres that contributed to this study. Moreover, we were not able to include an external validation for this work, as we did not have access to a comparable group from other institutions with CMR scans dating back to 2003. Nonetheless, internal validation with the bootstrapping method we used is considered a suitable and robust validation for prediction models^32^. Our aim was to identify predictors of survival from the time CMR was undertaken, thus we only included information from that period. Although some parameters may have changed in subsequently, we feel this change would have diluted rather than strengthened any associations seen. Furthermore, no other adverse events, including hospitalisation for heart failure, acute coronary syndrome, stroke were evaluated in this study. This is also important as we did not show a difference in mortality between men and women, although larger studies might reveal differences in cardiac hospitalisations. In addition, as this study started prior to our increased use of T1 mapping and appropriate quality assurance^33^ or indeed 4D flow^34^ thus, we were unable to incorporate such variables. Finally, with an increase in the use of transcatheter aortic valve replacement (TAVR) especially in low- and medium-risk populations^35^, it is uncertain whether this risk score will be valid in this population, or whether a different risk score needs to be designed. However, for the SAVR cohort, this score is robust and internally validated using the strongest statistical validation mechanism possible.

## Conclusion

In this large prospective registry-based study with the longest follow-up to date, we show that the risk of mortality in patients following SAVR remains high. We identify that the age of the patient at the time of SAVR, LVSV indexed, LAEF, presence of any myocardial fibrosis and use of antiplatelet therapy can be utilised to provide an estimate of mortality for such patients through a risk score, and help guide management both before and after surgery.

## Supplementary Information

Supplementary Information.

## Data Availability

All data can be obtained following reasonable request to the corresponding author.

## Abbreviations

ACE: Angiotensin converting enzyme
ARB: Angiotensin receptor blockers
AS: Aortic Stenosis
AVA: Aortic valve area
BSA: Body surface area
CABG: Coronary artery bypass grafting
CMR: Cardiovascular Magnetic Resonance
CVI: Cardiovascular Imaging
DM: Diabetes Mellitus
IQR: Interquartile range
LAEF: Left atrial Ejection Fraction
LGE: Late gadolinium enhancement
LV: Left Ventricle
LVEF: Left Ventricular Ejection Fraction
LVSV: Left ventricular stroke volume
NYHA: New York Heart Association
RVEF: Right ventricular Ejection Fraction
SAVR: Surgical aortic valve replacement
SD: Standard deviation
TAVR: Transcatheter aortic valve replacement
