# Supplementary material for: A novel cardiovascular magnetic resonance risk score for predicting mortality following surgical aortic valve replacement
Source: Sci Rep. 2021 Oct 12;11:20183. doi: 10.1038/s41598-021-99788-7 (PMC8511276; doi:10.1038/s41598-021-99788-7)
Supplement: Supplementary file 1 — Supplementary Information. [file 41598_2021_99788_MOESM1_ESM.docx]

**Supplementary material**

**A novel cardiovascular magnetic resonance risk score for predicting mortality following surgical aortic valve replacement**

**Authors:** Vassilios S Vassiliou^1,2*^ MBBS, PhD, FESC, FRCP, FACC Menelaos Pavlou^3^ BSc, PhD, Tamir Malley^1^ MBBS, Brian P Halliday^1*^ MBBS, PhD Vasiliki Tsampasian MSc, MD, MRCP^2^ Claire E Raphael^1^ Gary Tse^4^ MBBS, PhD, MBBS, PhD, Miguel Silva Vieira^1^ MD, Dominique Auger^1^ MD, PhD, Russell Everett^5^ MBBS, PhD Calvin Chin^5,6^ MD, PhD, Francisco Alpendurada^1^ MD, PhD, John Pepper, MD, MS^1^, Dudley J Pennell^1^ MD, PhD, David E Newby^5^ MD, PhD, Andrew Jabbour^7^ MD, PhD, Marc R Dweck^5#^ MD, PhD, Sanjay K Prasad^1#*^ MD, PhD, FESC

^1^ Department of CMR, Royal Brompton Hospital and National Heart and Lung Institute, Imperial College London

^2^ Norwich Medical School, University of East Anglia and Department of Cardiology, Norfolk and Norwich University Hospital, UK

^3^ Department of Statistical Science, University College London, UK

^4^ Second Hospital of Tianjin, Medical University, China

^5^ Centre for Cardiovascular Sciences, University of Edinburgh

^6^ Department of Cardiology, National Heart Centre, Singapore

^7^ Department of Cardiology, St. Vincent's University, Sydney, New South Wales, Australia.

^#^joint senior authors

*joint corresponding authors

**Corresponding authors**

Dr Vassilios Vassiliou

Floor 2, Bob Champion Building, Norwich Medical School,

James Watson Road, Norwich, NR4 7UQ, UK

Email: v.vassiliou@uea.ac.uk

Twitter: @vass_vassiliou

Dr Brian Halliday

CMR Unit, Royal Brompton Hospital

Sydney Street

London, SW3 6NP, UK

Email: b.halliday@rbht.nhs.uk

Prof Sanjay Prasad

CMR Unit, Royal Brompton Hospital

Sydney Street

London, SW3 6NP, UK

Email: [s.prasad@rbht.nhs.uk](mailto:s.prasad@rbht.nhs.uk)

**Methods**

*Missing data*

No predictor was missing more than 10% of data. Despite this, for predictors with any missing data, multiple imputation by chained equations (ICE) was used to impute the missing data. Logistic regression was used to identify the predictors of missingness and data were assumed to be missing at random. The outcome, the Nelson—Aalen estimate of the cumulative hazard, pre-specified potential predictors for the outcome, and predictors of missingness were included in the imputation model. Variable selection in the multiply imputed was performed using the ‘stack approach’. Coefficient estimates for the predictors retained in the final model were combined using Rubin’s rules ^1^. The performance measures were estimated in each imputed dataset; optimism-adjusted C-index and calibration slopes were calculated by also combining the estimates using Rubin’s rules ^2^.

1. Rubin D. Multiple Imputation for Nonresponse in Surveys. (Sons JW and, ed.). New York; 2004.

2. Wood AM, White IR, Royston P. How should variable selection be performed with multiply imputed data? Stat Med. 2008;27(17):3227-3246. doi:10.1002/sim.3177

Supplementary Material Figure 1


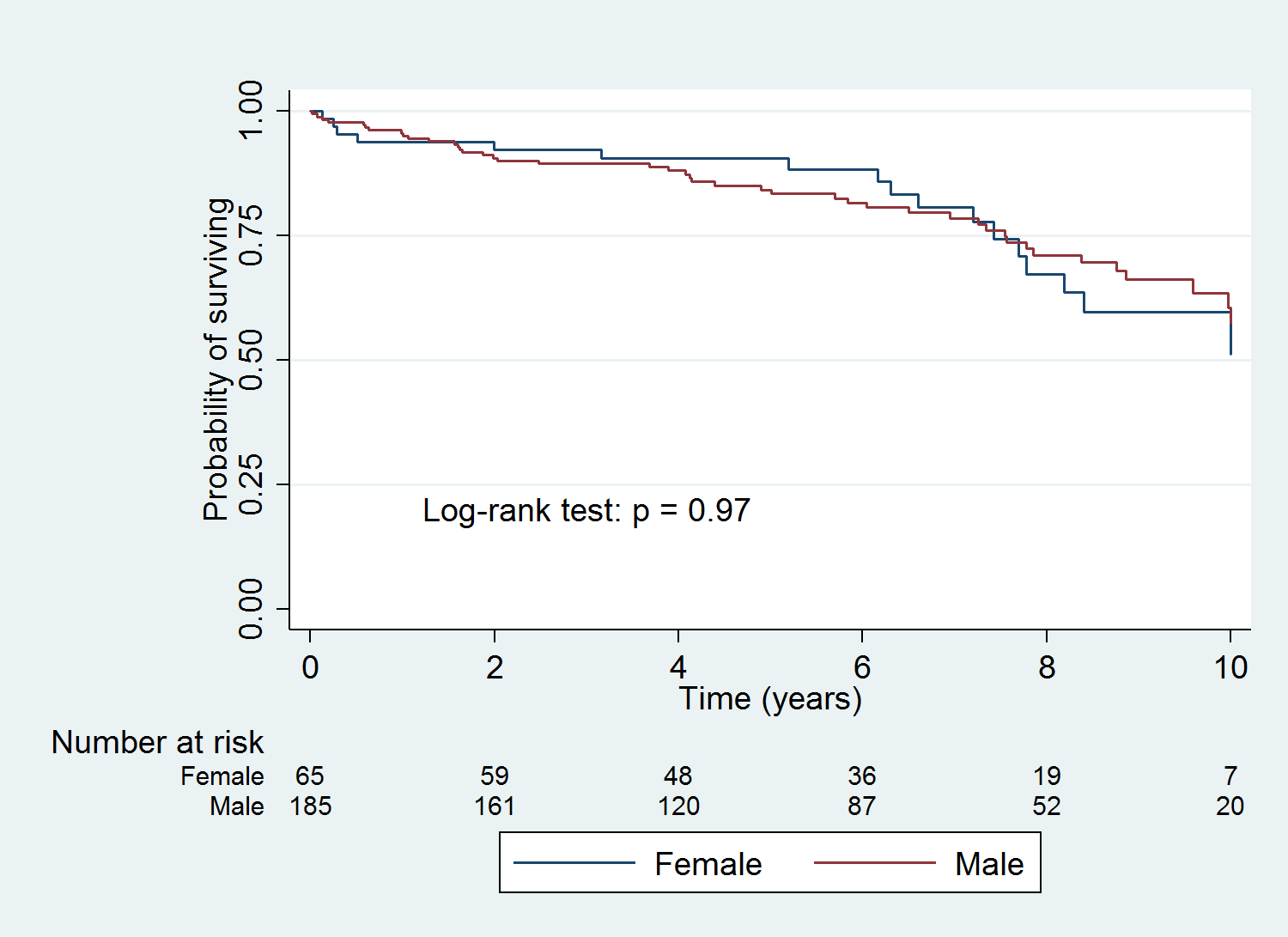


Legend Supplementary Material Figure 1: Survival following AVR between male and female patients, indicating no difference in outcome.
